# Increased Preference and Value of Consumer Products by Attentional Selection

**DOI:** 10.3389/fpsyg.2019.02086

**Published:** 2019-09-25

**Authors:** Nadiia Makarina, Ronald Hübner, Arnd Florack

**Affiliations:** ^1^Department of Psychology, University of Konstanz, Konstanz, Germany; ^2^Graduate School of Decision Sciences, University of Konstanz, Konstanz, Germany; ^3^Department of Psychology, University of Vienna, Vienna, Austria

**Keywords:** selective attention, value-based decision, choice-induced preference change, consumer decision, visual search

## Abstract

It is usually assumed that individuals base their preferences for products or other items on the utility or value associated with the items. However, there is evidence that the attentional selection of an item alone already modulates the preference for that item. This has been shown, for instance, in preference choice tasks with unknown consumer products. Products that served as targets in a preceding visual search task were preferred to former distractor products. However, it is unclear whether such effects can also be observed when individuals have pre-existing attitudes toward products and whether attentional selection can change the perceived value of products. Hence, the aim of the present research was to replicate the attentional-selection effect on choice with known products and examine whether selective attention affects the perceived value of products beyond choosing the items. In two experiments, we replicated the attentional-selection effect on item preference in a choice task. Items that had served as targets in the search task were preferred to previous distractors. Introducing a response deadline in the preference-choice task in Experiment 2 did not further increase this effect. However, the value of former targets was rated higher than that of former distractors. Hence, the present results indicate that attentional selection not only affects preference choices but can also increase the value of attended and selected items.

## Introduction

Many theories of decision-making assume that the choice between different objects is based primarily on the objects’ values, which are the result of corresponding benefits (utility, incentives, good feelings, great taste, etc.) experienced or expected by the persons (Brosch and Sander, 2013). However, there is increasing evidence that the values reflected by these *value-based decisions* are not only determined by previous benefits but also by other factors. For instance, it has been observed that we not only choose what we prefer, but also prefer what we chose. An early example is the study by Brehm (1956), who applied a free-choice paradigm in which participants first indicated their preference for several items, then made binary choices between pairs of equally preferred items, and finally indicated their preferences for the items again. As a result, the chosen items increased in value compared to the non-chosen ones, whereas the non-chosen items decreased in value. Meanwhile, this outcome has been replicated many times (Izuma et al., 2010; Sharot et al., 2012; Coppin et al., 2014; Salti et al., 2014; Koster et al., 2015).

However, a problem in such studies of choice-induced preference or value changes is to disentangle the different effects that influence the valuations. For instance, it is possible that preference ratings increase simply due to regression toward the mean after choice. That is, if valuations of items are only noisy representations of underlying preferences, then sequential valuations can be considered as independent draws from the same distribution. It has been shown that in this case, the observed spread in the second valuation tends to be closer to the true preferences than the first one (Chen and Risen, 2010; Izuma and Murayama, 2013). Therefore, Voigt et al. (2017) used a control-group design to prevent such regression effects and found that choice can indeed change preferences.

Interestingly, preferences and corresponding values can not only be modified by value-based decisions but also by decisions completely unrelated to preferences. What is sufficient is the involvement of selective attention. In corresponding studies, participants have first to select a predefined target among distractor items. In such tasks, selective attention is important to process relevant information while suppressing the processing of irrelevant information (e.g., Hübner et al., 2010). Irrelevant stimuli must be suppressed, because they can compete for response control. The strength of this competition depends on learned stimulus-response associations as well as on the value of the items (e.g., Dummel and Hübner, 2017).

If, after a selective-attention task, participants have to choose between previously selected or ignored items, they prefer the former ones. Moreover, this change of preference is likely due to value changes. One phenomenon in this respect is *distractor devaluation* (Fenske and Raymond, 2006). Raymond et al. (2003), for instance, found that previously ignored stimuli were evaluated more negatively than previously attended or novel stimuli. They assumed that task-irrelevant items competing for response control are inhibited, and that this inhibition is remembered when these items have later to be evaluated, which leads to affective devaluation. Meanwhile, distractor devaluation has been observed in various studies (e.g., Kiss et al., 2007; Martiny-Huenger et al., 2014). However, the effect does not always show up reliably. In the study by Martiny-Huenger et al. (2014), for instance, distractor devaluation was not significant in the first experiment. Rather, target evaluation was marginally significant. In other studies on distractor evaluation, neutral stimuli were not even included (e.g., Kiss et al., 2007).

Whereas these studies demonstrate mainly distractor devaluations, there is at least one research paper in which also positive effects for selected items have been reported. In Janiszewski et al. (2013), participants first performed a preview-search task, in which they had to decide as fast as possible, which one of two presented items matches a previously shown target. When the participants had to choose later between seen items, they more often preferred a previously selected item to a neutral one, and a neutral item to a previously ignored one. Janiszewski et al. (2013) called these effects *mere-selection* and *mere-neglect* effect, respectively.

Thus, whereas various studies show mostly negative effects of selective attention on preference, Janiszewski et al. (2013) also found positive effects. This could indicate that attentional selection can also increase the value of selected items. However, because the study is special in several respects, it is open to what extent their basic results can be generalized. Therefore, the aim of the present study was to replicate part of Janiszewski et al. (2013) results with different stimuli and a different procedure.

One specificity of Janiszewski et al. (2013) study is that pictures of consumer products were used as stimuli. Because consumer products are usually already associated with a value, it is rather difficult to detect the relatively small choice-induced preference changes. If at all, then such changes are most likely observed for choices between items that initially had similar values. Janiszewski et al. (2013) tried to avoid initially different values by using products unknown to the participants. However, it might be challenging or, at least less relevant, for participants to choose between products that they had never encountered before and that they will not be able to purchase in the future. Therefore, participants might have applied the simple heuristic to choose the item that they had previously selected. Thus, although Janiszewski and colleagues speculate that their results generalize to products from the supermarket, this remains to be shown. Therefore, in our study, we used images of known products as stimuli. Similar to the research by Janiszeweski and colleagues, our experiments also started with a visual-search task, but instead of searching for a specific pre-viewed item of unknown products, our participants had to search among known products for the item of a pre-specified category. The search task was then followed by a preference-choice task in which on each trial the participants saw two items of the *same category*. One item in each pair previously served as target, whereas the other served as distractor. Importantly, the items in each pair were of similar previous value. For assessing the previous value of the items, liking ratings were collected in an independent preliminary study. If attentional selection of an item increases its preferability, then former targets should be chosen more frequently in the preference-choice task than former distractors.

To differentiate positive from negative preference changes, Janiszewski et al. (2013) used neutral items, which were not part of the search display but were shown as often as target and distractor items. An equal presentation frequency is necessary, because stimuli that had been shown more frequently could be preferred to novel ones just because of a mere exposure effect (Zajonc, 1968). Strictly speaking, equal presentation times are not sufficient for controlling mere exposure. What actually needs to be equalized are the inspection times, which has been done by Florack et al. (2019). However, even if mere exposure effects can be excluded, there was still a confound. Neutral items were always shown alone, whereas each distractor item always occurred together with a target item. Moreover, each target was shown together with a distractor in the search display as well as alone in the preview-search display. Thus, it seems impossible to simultaneously avoid confounds with respect to stimulus frequency and those with respect to stimulus context. In view of these difficulties, we decided to use only target and distractor items and restrict our goals to show that preference changes can also be induced by choices between known products and that attentional selection can lead to changes in value beyond choice.

Indeed, it must be noted that the applied method in Janiszewski et al. (2013) is not sufficient to conclude that the observed preference changes were due to value changes. Effects in a subsequence preference choice task on proportion can simply be due to learning of a response. That is, participants may choose the target more frequently than the distractor, simply because they have done so before in the visual-search task. Moreover, participants may have learned to attend to the target, which is known to increase choice proportion (Krajbich et al., 2010; Krajbich and Rangel, 2011). Finally, focusing on a target stimulus might result in considering this stimulus as a default option, which is chosen when preferences or options are similar or indifferent, and when there is no motivation to switch (Gal, 2006).

Because it is important to know whether preference changes are due to value changes, we also asked our participants to rate the items with respect to liking. If the ratings of the targets were higher compared to those of the distractors, then this would indicate that the values had also changed.

Thus, with our method, we avoided confounds with spatial stimulus isolation, extended the investigation to known consumer products, and were able to assess whether selective attention not only changes preferences but also values. We used consumer products in our research, because participants make value-based choices between consumer products every day and have pre-existing attitudes toward consumer products. Thus, compared to arbitrary shapes or color patterns, products possess meaning for consumers, and finding attentional-selection effects for known products would show that these effects could have a relevance for consumer decision-making in real life. Indeed, consumers are exposed to visually complex environments in many supermarkets and online shops, where they selectively attend to some products and neglect others. Furthermore, marketing applies tools to create selective attention, for example, by shelf placements (Atalay et al., 2012) or gaze cueing (Palcu et al., 2017). Finally, selective attention could be easily implemented as advertising in computer games. It is highly relevant for the advertising industry to know whether selective attention might increase preferences more than simple exposure.

## Experiment 1

The method in our first experiment was similar to that in Janiszewski et al. (2013). However, instead of unknown products, we presented images of products that were mostly familiar to the participants. Moreover, we used a categorical choice as task. In a pair of items, our participants simply had to select the member of the category (sweet or savory snack) that had been pre-specified for the given block of trials. Because the distractor was always a member of the other category, the target did not have to be shown before the search display on each trial. Therefore, no items were shown alone.

For the subsequent preference-choice task, items of the same category and with similar previous value were paired. The previous values were taken from a preliminary independent rating study. In a final step, the participants also had to rate the items with respect to liking. Thus, if attentional selection has an effect, then the preference for targets should be higher than for distractors. Moreover, if value is also affected, then the corresponding ratings should differ accordingly.

### Method

#### Preliminary Value Rating Study

Fifty-two volunteers (19–65 years, *M* = 27.3, SD = 9.54, 29 females) were recruited at the University of Konstanz for participating in the rating study. For their participation, they received € 8 or course credit, and were offered to choose one out of a set of snacks they had previously seen on the screen during the experiment. The task was to rate 77 pictures of different snacks shown one after another on the computer screen. By horizontally moving a curser with the arrow keys on the keyboard, they had to indicate on a 5-point Likert scale ranging from 1 (“don’t like it at all”) to 5 (“like it very much”) how much they liked the snacks.

#### Participants

For an expected small to medium effect size of 0.4, *α* = 5%, and *β* = 20%, a power analysis revealed a desired sample size of 41. By also taking possible dropouts into account, 46 students from the University of Konstanz were recruited *via* an online recruitment system (ORSEE, Greiner, 2015) for participating in the experiment (37 females, aged from 18 to 32 years, *M* = 22, SD = 3.0). For their participation, students received either € 10 or course credit. This study was carried out in accordance with the recommendations of the ethical guidelines of our University’s IRB (Ethics Committee) with written informed consent from all subjects. All subjects gave written informed consent in accordance with the Declaration of Helsinki.

#### Stimuli

Sixteen pictures of sweet snacks (e.g., biscuits, chocolates, wine gums, and candy bars) and 16 pictures of savory snacks (e.g., peanuts, potato chips, pretzel sticks, and smoked almonds) were selected from the preliminary value-rating study as stimuli for this experiment. With respect to the planned preference-choice task, the selection criterion for these items was that all should have a similar mean value. For the selected 16 sweet and 16 savory items, the average value was 1.98 (SD = 0.375) and 2.00 (SD = 0.366), respectively, on the 5-point scale (see above).

For the preference-choice task, we needed pairs of items from the same category, where one item served as target in the search task, and the other as distractor. For this objective, the set of pictures for each category was divided into two subsets: A_a_ and B_a_ for the savory items, and A_e_ and B_e_ for the sweet items.

All pictures had an extension of 250 × 250 pixels on a 19″-monitor with a resolution of 1,280 × 1,024 pixels. The pictures subtended a visual angle of 8.15° at an approximate viewing distance of 45 cm. For the search task and the preference choice task, the items were shown as pairs. One item was presented on the left side of the screen, and another on the right side. The distance from the center of each picture to the screen center measured 3.84° of visual angle.

#### Search Task

For the search task^1^, the items in the subsets were also combined pairwise. Specifically, each of the eight items in set A_a_ of savory snacks was paired with each of the eight items in set A_e_ of sweet snacks, resulting in 64 item pairs. In the same manner, 64 pairs were constructed from the B subsets. Half of the participants had to search in A pairs (A_a_, A_e_) for savory items and in B pairs (B_a_, B_e_) for sweet items. For the other half of participants, the mapping between pair type and target category was reversed. As consequence, each item serving as target for one group of participants, served as distractor for the other group, and vice versa. Together, each item occurred eight times as target, and eight times as distractor.

This two-group design was necessary to control for effects of stimulus differences. Although the stimuli were matched with respect to their initial value, there are other potential confounds. For instance, it could be that in one group, the target items in both stimulus sets have a higher perceptual saliency than the distractors. Then, they might not only be found very fast, their higher saliency might also favor their selection in the preference-choice task, either directly or due to memory effects (Santangelo, 2015). With a second group of participants for which the roles of targets and distractors are reversed, we can control for such biases of stimulus features, because, across participants an item serves as a target as often as a distractor. Consequently, differences in choice behavior cannot be attributed to low-level stimulus features.

**Figure 1:**
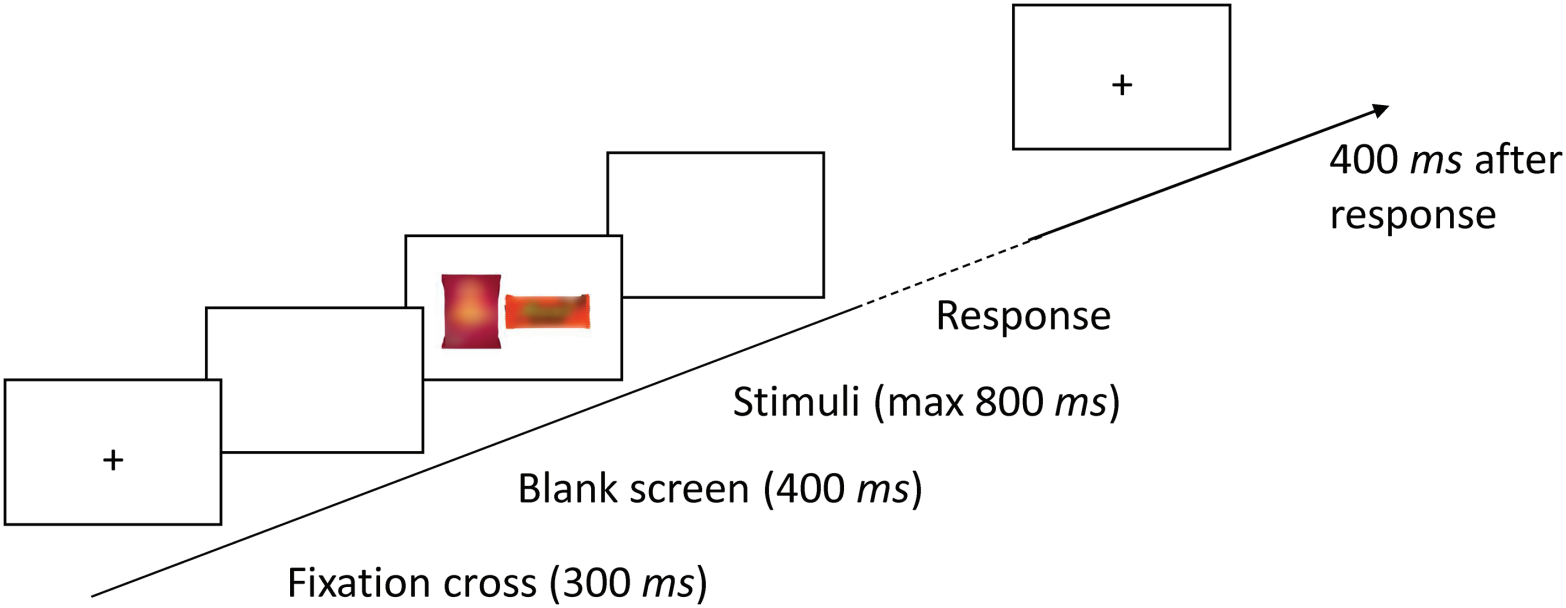
Sequence of an example trial of the visual search task in Experiments 1 and 2. The stimlus items are blurred in this example for copyright reasons.

Each trial started with a fixation cross, presented for 300 ms at the center of the screen (see Figure 1). After a blank screen presented for 400 ms, the pair of snacks (one sweet, the other savory) appeared and remained visible until the participant’s response, but no longer than 800 ms. The task was to indicate the position (left or right) of the item belonging to the target category (sweet or savory) by pressing the corresponding mouse button. Errors were signaled by a 100 ms tone (1,000 Hz). Target category was blocked (64 trials per block). Block order and stimulus subsets (A pairs or B pairs) were counterbalanced across participants. Thus, altogether, there were 128 trials for each participant.

#### Preference Choice Task

Participants performed the preference-choice task shortly after the search task. As stimuli served 16 pairs of snacks, which were shown to all participants. Each pair consisted of two items from the same category (e.g., two sweet snacks), where one item was taken from subset A and the other from subset B. For instance, pairs of sweet snacks had the form (A_e_, B_e_). The pairs were constructed in such a way that the value of the two items in each pair was as equal as possible according to the mean ratings from the preliminary rating study. The mean of the absolute value difference between the items in a pair was 0.038 (SD = 0.0009).

Importantly, depending on the person, one item in each pair had previously been a target (distractor) in the search task, whereas the other had been a distractor (target). Thus, by using two groups of participants with reversed mappings between stimulus sets and item role, each item was a target in one group as often as a distractor in the other group. By this procedure, possible confounds due to saliency differences were prevented.

Participants had to choose for each item pair which snack they preferred. Position of the items in a pair and order of pairs were randomly determined for each participant. The overall procedure was similar to that of the search task, except that the stimuli remained visible until response. There was no time limit for responding.

#### Value Ratings

At the end of the experiment, that is, after the search task and the preference choice task, participants were asked to rate each item presented in the experiment with respect to their individual preferences on a 5-point Likert-scale from “do not like it at all” (0) to “like it very much” (4). The central position (2) was labeled “I don’t know the item.” The items were sequentially presented on a computer screen with the rating scale placed under the stimulus. The order of the items was randomized.

### Results

#### Search Task

##### Response Times

The data of three participants were excluded from analyses because of their low accuracy rate (<60%). Furthermore, response times (RTs) smaller than 100 ms and larger than 2.5 standard deviations of the RTs were excluded from analysis (<2.5% of all data). Mean RT was 527 ms (SD = 71.3 ms). The RTs of correct responses were subjected to an analysis of variance (ANOVA) with the between-participants factor *item set* (A vs. B) and the within-participant factor *target category* (savory snack vs. sweet snack). For all statistical analyses, the GNU-software R (version 3.3.1) was used.

The factor *target category* had a significant effect, *F*(1, 41) = 8.90, *p* < 0.01, $\eta_{p}^{2}$ = 0.178. However, there was also a significant interaction between *target category* and *item set*, *F*(1, 41) = 6.66, *p* < 0.05, $\eta_{p}^{2}$ = 0.140. Savory snacks were identified faster than sweet snacks, but only for item set B (see Table 1). The interaction was presumably due to visual feature differences between the savory and sweet items in set B.

**Table 1:** Results of the search task in Experiment 1.

Item set	Target category	Mean RT (ms)	Error rate (%)
A_a_	Savory	533 (82.6)	6.40 (4.35)
A_e_	Sweet	536 (71.4)	6.01 (3.51)
B_a_	Savory	494 (67.1)	3.36 (2.63)
B_e_	Sweet	548 (54.9)	5.99 (2.63)

*The values in parenthesis are the standard deviations*.

##### Error Rates

Mean error rate was 5.42%. The rates were subjected to an ANOVA analogous to that for the RTs. The factor *target category* was significant, *F*(1, 41) = 4.71, *p* < 0.05, $\eta_{p}^{2}$ = 0.103, as was the interaction between the two factors, *F*(1, 41) = 8.01, *p* < 0.01, $\eta_{p}^{2}$ = 0.163. As can be seen in Table 1, whereas the error rates were similar for the target categories in set A, fewer errors occurred for savory snacks in set B (see Table 1).

#### Preference Choice Task

**Figure 2:**
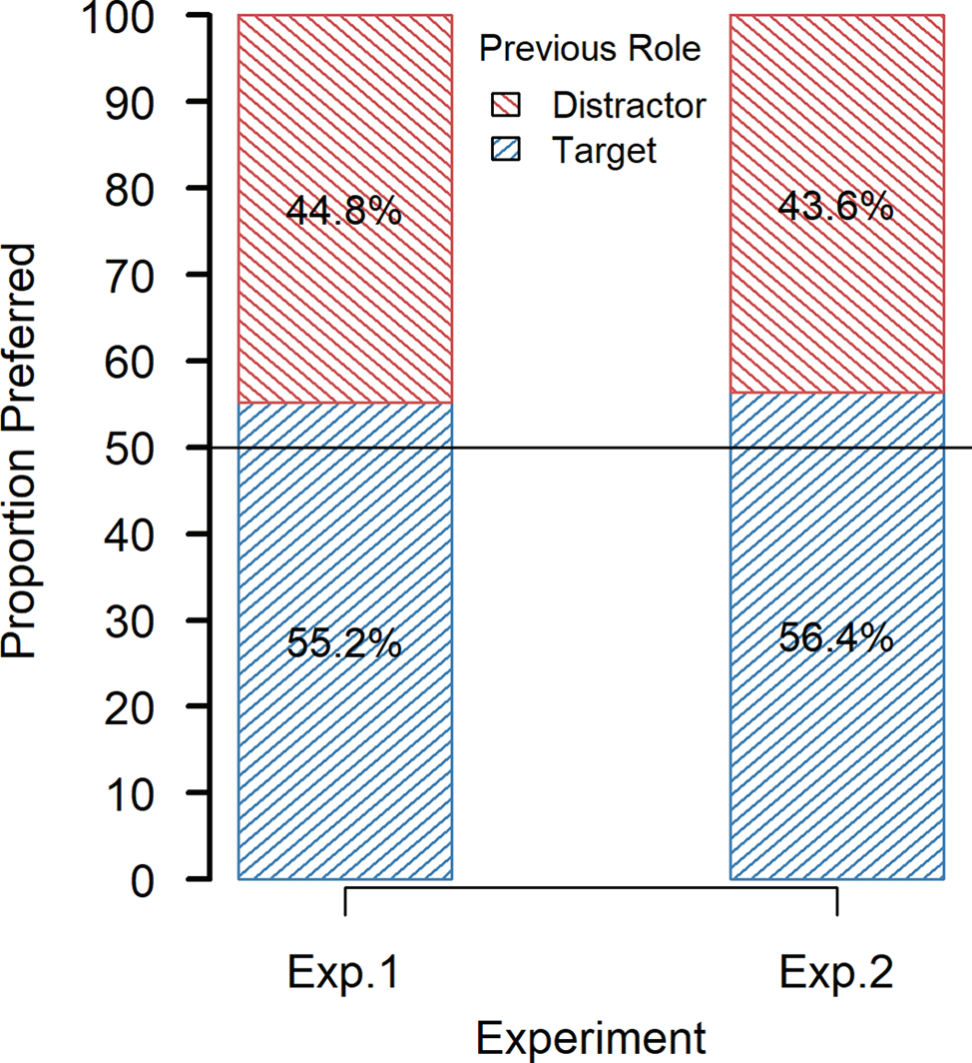
Choice proportions in the preference-choice task in Experiments 1 and 2.

In the preference choice task, snacks were preferred on 55.2% of the trials when they served as targets, and only on 44.8% when they served as distractors (see Figure 2). A *t*-test (one-sided) for the difference in choices revealed a significant deviation from chance, *t*(42) = 2.00, *p* < 0.05, Cohen’s *d* = 0.305.

We also analyzed the RTs for the preference choices. A *t*-test revealed a significant effect, *t*(42) = 2.20, *p* < 0.05, *d* = 0.335. Choices were faster for former targets than for former distractors (*M* = 1,091 ms, SD = 495 ms vs. *M* = 1,209 ms, SD = 567 ms).

#### Value Ratings

**Figure 3:**
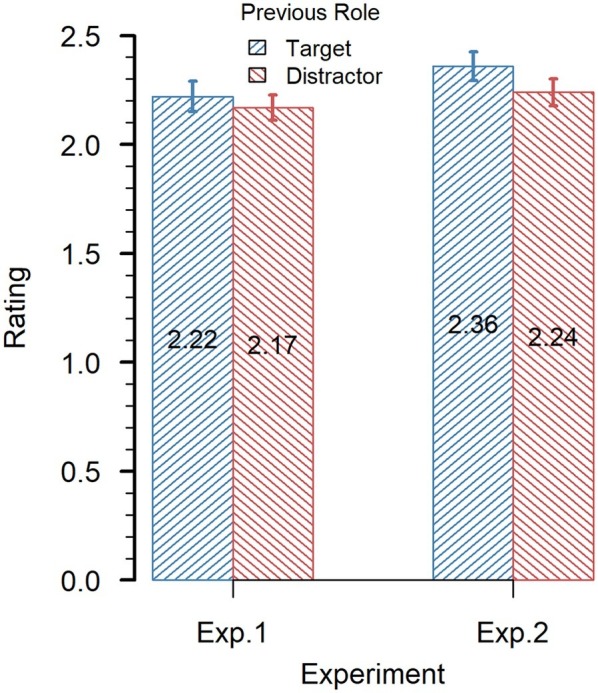
Value ratings of the products in Experiments 1 and 2. The error bars represent the standard error of the mean.

The mean rating for previous targets was numerically higher than that for previous distractors, (*M* = 2.22 ms, SD = 0.639 ms vs. *M* = 2.17 ms, SD = 0.540 ms, see also Figure 3). However, a *t*-test (one-sided) revealed that the difference was not significant, *t*(42) = 0.789, *p* = 0.217, *d* = 0.120. Further analysis shows that, on average, for 4 of the 32 items the central (“I do not know the item”) position was chosen. To test whether former targets and distractors differed in value for known items, we repeated the analysis after removing the data of these trials. It revealed practically identical results.

Also, we compared the mean ratings of the target and distractor products to the mean rating obtained in the preliminary study (*M* = 1.99 ms, SD = 0.365 ms). The analyses revealed that, in the present study, the target ratings were significantly higher than in the preliminary study, *t*(42) = 3.73, *p* = 0.001, Cohen’s *d* = 0.569, as were the distractor ratings, *t*(42) = 3.84, *p* = 0.001, *d* = 0.585.

### Discussion

The results of our experiment show that forced selection in a visual search task increases the likelihood that in a subsequent preference-choice task previously selected items will be preferred (see Figure 2). Thus, we not only replicated the attentional selection effects found by Janiszewski et al. (2013) but also generalized their results obtained with unknown products to choices between known products for which participants already had established preferences. In the preference-choice task, we also found that previous target items were chosen faster than previous distractor items.

A prerequisite for detecting small selection-induced changes of value by means of a preference-choice task is to have pairs of items of similar value. Our results demonstrate that it is not necessary to construct such pairs by using unknown products. Rather, equally valued items can also be paired using mean liking ratings.

The hypothesis that attentional selection also increases the item’s value could not be confirmed with our data. Although the mean rating was numerically higher for previous targets than for previous distractors (see Figure 3), the difference was not significant. Compared to the value ratings from the preliminary study, value ratings in the main experiment were significantly higher for both previous targets and previous distractors. This could not only be an effect of mere exposure (Zajonc, 1968) but also due to context effects, because the set of products was much larger in the preliminary study.

Although the attentional selection effects were significant in the preference-choice task, they were relatively small. Therefore, we conducted a further experiment in which we examined whether time pressure during choice increases the size of the effect.

## Experiment 2

The results in the previous experiment show that former targets were preferred more frequently in the preference-choice task than former distractors. Unfortunately, this effect was relatively small. However, there was a strong effect in choice speed. Preference choices were much faster for targets than for distractors. This could indicate that the decisions largely relied on automatic processes, which are usually faster than controlled ones (Hübner et al., 2010). Choosing previous distractors, in contrast, may have relied more on deliberate processes, which are usually relatively slow. This explanation is also in line with results of Pieters and Warlop (1999), who found that under time pressure consumers preferably process information that requires less cognitive effort and ignore cognitively more demanding information.

Based on these results and ideas, we tried in the present experiment to transfer at least part of the effect in the RTs to the effect in choice proportions by implementing a deadline in the preference-choice task. Accordingly, the present experiment was similar to the previous one, except that the participants decided under time pressure which item they preferred.

### Methods

#### Participants

Based on a similar power-analysis as in Experiment 1, 48 students from the University of Konstanz were recruited *via* an online recruitment system (ORSEE, Greiner, 2015) for participating in the experiment (38 females, age from 18 to 30 years, *M* = 22, SD = 3). For their participation, students received either € 10 or course credit.

#### Procedure

We used the same stimuli and a similar procedure as in Experiment 1. Different from the previous experiment, participants had to respond before a 700-ms deadline in the preference-choice task. Participants were told that only choices that meet the deadline are considered as successful. After each trial, participants received feedback about how successful their performance was. In case they met the deadline, they saw a full shopping cart as clip art and a smiley shortly after the trial. If they responded too slowly, a clip art of a supermarket and a frowny was presented. As in Experiment 1, the participants were finally asked to rate all snacks on a Likert scale. Because in the previous experiments excluding trials on which the central position (“I don’t know the item”) was chosen had no effect, this specific labeling was dropped in the present experiment.

### Results

#### Search Task

The data of one participant was excluded from all analyses because of the low accuracy rate (<60%). One further participant was excluded from RT analyses, because of the extremely long RTs (*M* = 2,209 ms). Furthermore, individual response times (RTs) smaller than 100 ms and larger than 2.5 standard deviations of the RTs were excluded from analysis (<2.8% of all data). Mean RT was 532 (SD = 75.0). The RTs of correct responses were subjected to an analysis of variance (ANOVA) with the between-participants factor *item set* (A vs. B) and the within-participant factor *target category* (sweet snack vs. savory snack). The factor *target category* had a significant effect, *F*(1, 44) = 11.8, *p* < 0.001, $\eta_{p}^{2}$ = 0.212. Savory snacks were identified faster than sweet snacks (*M =* 511 ms, SD *=* 59.6 ms vs. *M =* 553 ms, SD = 83.2 ms). The overall pattern of results was similar to that in Experiment 1. However, this time the interaction between the two factors was not significant, *F*(1, 44) = 1.04, *p* = 0.31, $\eta_{p}^{2}$ = 0.023 (see also Table 2). Savory snacks were also identified faster than sweet snacks in item set A.

**Table 2:** Results of the search task in Experiment 2.

Item set	Target category	Mean RT (ms)	Error rate (%)
A_a_	Savory	531 (54.4)	6.87 (6.54)
A_e_	Sweet	560 (85.6)	5.66 (4.25)
B_a_	Savory	492 (59.4)	3.76 (2.50)
B_e_	Sweet	546 (43.5)	8.74 (6.67)

*The values in parenthesis are the standard deviations*.

The mean error rate was 6.26%. The error rates were subjected to an ANOVA that was analogous to that of the RTs. The factor *target category* was significant, *F*(1, 44) = 4.45, *p* < 0.05, $\eta_{p}^{2}$ = 0.092. However, there was also a significant interaction between target category and item set, *F*(1, 44) = 12.0, *p* < 0.01, $\eta_{p}^{2}$ = 0.214. It was similar to that in Experiment 1 (see Table 2).

#### Preference Choice Task

In the preference choice task, previous target items were chosen on 56.4% of the trials (see Figure 2), which is significantly greater than chance, *t*(47) = 3.05, *p* < 0.01, *d* = 0.440. A *t*-test showed that target choices were not significantly faster than distractor choices (*M* = 542 ms, SD = 105 ms vs. *M* = 576 ms, SD = 209 ms), *t*(47) = 1.42, *p* = 0.162, *d* = 0.205. The participants missed the deadline on 12% of the trials. Excluding these data from analysis did not change the results (56.7% of targets chosen).

#### Value Ratings

This time, the mean value rating for previous targets (*M* = 2.36 ms, SD = 0.644 ms) was significantly higher than that for previous distractors (*M* = 2.24 ms, SD = 0.611 ms), *t*(46) = 2.23, *p* < 0.05, *d* = 0.325, (see also Figure 3). Both mean ratings were again significantly higher than the ratings from the preliminary study (targets: *t*(46) = 5.25, *p* = 0.001, *d* = 0.766; distractors, *t*(46) = 3.50, *p* = 0.01, *d* = 0.511).

A further analysis with the pooled value ratings of both experiments also revealed that former targets (*M* = 2.29 ms, SD = 0.444 ms) were rated significantly higher than former distractors (*M* = 2.20 ms, SD = 0.395 ms), *t*(89) = 2.18, *p* < 0.05, *d* = 0.230.

### Discussion

The results from this experiment again replicated the attentional-selection effect. Different from our expectation, introducing a deadline increased the proportion of selected targets only slightly relative to the previous experiment (56.4 vs. 55.2%); the corresponding effect sizes were (0.440 vs. 0.305). A comparison revealed that the difference in proportion between the experiments was not significant, *t*(82.7) = 0.343, *p* = 0.733.

Concerning the value ratings, former target items were rated higher in value than former distractors. Numerically, this was already the case in the previous experiment. Moreover, for the pooled data of both experiments, the difference was also significant. In the present experiment, however, the effect was stronger and significant. Because the search task was similar to that in Experiment 1, but the preference-choice task differed, it seems that at least part of the value was induced by the time pressure during preference choices.

## General Discussion

Based on the observation that people not only choose what they prefer but also prefer what they chose, there is growing interest in the mental mechanisms behind such choice-induced preference changes. Interestingly, not only previous preference choices but also attentional selection in general seems to modulate preferences. A prominent effect in this respect is *distractor devaluation* (Fenske and Raymond, 2006), i.e., the phenomenon that the values of stimuli, which had to be ignored in a previous visual-search task, are judged to be less than the values of previously selected stimuli. Whereas distractor devaluation has mainly been observed with abstract stimuli, Janiszewski et al. (2013) used images of consumer products and found not only decreased preferences for ignored items but also enhanced preferences for selected stimuli.

Although the results of Janiszewski et al. (2013) are promising and in line with observations from marketing research that increased visual attention on a product increases the product’s preference (Chandon et al., 2009; Palcu et al., 2017), they are nevertheless limited. The reason is that, for constructing stimulus pairs for the preference-choice task whose items were originally of equal preference, the researchers used products that were unknown to the participants. Clearly, preferences for unknown products should initially not differ systematically. However, choosing between unknown products has little ecological validity and corresponding results might therefore not be generalizable to choices between known products. Therefore, the aim of the present study was to replicate the observed selection-induced preference effects with known products for which the participants had already developed some preferences. For constructing pairs of equal-preference products, we relied on the value ratings obtained in a preliminary study.

In Experiment 1, we replicated the attentional-selection effects observed by Janiszewski et al. (2013). Former targets in the search task were preferred to former distractors in the preference task. Unfortunately, the effect was relatively small in the choice proportions. However, former targets were also chosen much faster than former distractors. This speed advantage could indicate that choosing former targets was less effortful than choosing former distractors. Due to their previous selection, targets could have automatically activated a response toward them in the preference-choice task. Therefore, to transfer at least part of the effects in RT to effect in choice proportion, participants in Experiment 2 had to respond before a deadline in the preference-choice task. Different from our expectation, preference was only numerically increased. Nevertheless, although time pressure did not have the expected effect, we reliably produced selection-induced preference changes for known products in two experiments.

Because we did not use neutral items, we cannot tell whether the preference changes were due to mere selection, to mere neglect, or to both. However, in three experiments, Florack et al. (2019) were recently unable to replicate mere-neglect effects. In view of this failure to show mere-neglect effects, it is likely that also in our experiments merely target preferences increased.

In addition to the limited generalizability to known products, it also remained open in the study of Janiszewski et al. (2013) whether the observed preference changes were related to value changes. To approach this question, we asked our participants to rate the value of the presented items at the end of the session. Because the products were rated after both the search task and the preference-choice task, effects cannot easily be attributed to the one or the other task. In our first experiment, there was no significant modulation of value, although it pointed in the expected direction. However, in Experiment 2, value ratings were higher for previous targets than for previous distractors. Pooling the data of both experiment also revealed a significant effect. In any case, the effect was more reliable in our second experiment. Because the main difference between the experiments was the time pressure in the preference task, this result suggests that at least part of the value modulation was caused by the choices in the preference task.

Finally, it should also be noted that the mean values for targets as well as for distractors were significantly higher than the mean value in the preliminary study. Reasons could be the mere exposure effect (Zajonc, 1968), or the fact that the context differed, i.e., the set of items in the experiments was reduced compared to the preliminary study.

Taken together, our results show that the instructed attentional selection of known consumer products in a search task changes the previously evolved preferences of the products. Selected items have a higher likelihood to be preferred on later occasions than ignored items, although the selection was instructed. Moreover, our results show that attentional selection also increases the relative value of the selected item, even if possibly mediated by later preference choices. What our results cannot show, though, is whether attentional selection increased the preference of targets, decreased the preference of distractors, or both. For distinguishing between these cases, we would also have needed neutral items. We have refrained from using neutral stimuli, because their definition is difficult, and this differentiation was not in the focus of our study.

Even if our experiments focused on basic effects of selective attention on preferences and values, in concert with the previous studies on effects of selective attention on consumer judgments and behavior (Janiszewski et al., 2013; Florack et al., 2019), they bear practical implications for marketing contexts. Indeed, the finding that the values and preferences for known products can be altered by selective attention beyond mere exposure, questions the simple marketing rule that every exposure is a good exposure. Such thinking in marketing is obvious, for example, in pricing models for advertising (Bolland, 1989). However, in-game advertising, for instance, provides huge opportunities to assign brands to targets or distractors. The present findings imply that such manipulation could have effects on preferences for perceived value of products. Because previous research has also shown that devaluation effects are sometimes, but not always shortlived (Serfas et al., 2017), we regard it as a fruitful approach for future research on consumer behavior to examine whether and under which circumstances effects of selective attention are enduring and can be applied in advertising.

## Data Availability

The raw data supporting the conclusions of this manuscript will be made available by the authors, without undue reservation, to any qualified researcher.

## Ethics Statement

This study was carried out in accordance with the recommendations of the ethical guidelines of our University’s IRB (Ethics Committee) with written informed consent from all subjects. All subjects gave written informed consent in accordance with the Declaration of Helsinki. According to our University’s IRB guidelines, the study is exempt from protocol approval, because it was not in any way harmful or invasive, without deception, and did not induce stress in participants.

## Author Contributions

RH, AF, and NM conceived the study. NM contributed stimuli, collected all the data, and wrote a first draft of the paper. NM and RH analyzed the data. AF contributed to the writing of the paper. RH revised the paper and supervised the study at all stages.

### Conflict of Interest Statement

The authors declare that the research was conducted in the absence of any commercial or financial relationships that could be construed as a potential conflict of interest.

## Footnotes

^1^One might question that our task with only two items in the display is indeed a search task. However, for comparability reasons, we adopted the naming from Janiszewski et al. (2013).
